# A systematic study of the N-glycosylation sites of HIV-1 envelope protein on infectivity and antibody-mediated neutralization

**DOI:** 10.1186/1742-4690-10-14

**Published:** 2013-02-06

**Authors:** Wenbo Wang, Jianhui Nie, Courtney Prochnow, Carolyn Truong, Zheng Jia, Suting Wang, Xiaojiang S Chen, Youchun Wang

**Affiliations:** 1 Department of Cell Biology, National Institutes for Food and Drug Control, No. 2 Tiantanxili, Beijing, 100050, P. R. China; 2 Department of Molecular and Computational Biology, University of Southern California, Los Angeles, CA, 90089, USA; 3 Chemistry Department, University of Southern California, Los Angeles, CA, 90089, USA; 4 Norris Cancer Center, University of Southern California, Los Angeles, CA, 90089, USA

**Keywords:** HIV, N-linked glycosylation site, Pseudovirus, Neutralization antibodies

## Abstract

**Background:**

Glycans on the human immunodeficiency virus (HIV) envelope glycoprotein (Env) play an important role in viral infection and evasion of neutralization by antibodies. In this study, all 25 potential N-linked glycosylation sites (PNGS) on the HIV-1 CRF07_BC Env, FE, were mutated individually to study the effect of their removal on viral infectivity, virion production, and antibody-mediated neutralization.

**Results:**

Removal of specific N-glycosylation sites has a significant effect on viral infectivity and antibody-mediated neutralization phenotype. Six of these glycosylation mutants located on the V1/V2 and C1/C2 domains lost infectivity. PNGS mutations located on V4/C4/V5 (except N392 on V4), were shown to increase viral infectivity. Furthermore, FE is much more dependent on specific glycans than clade B Env YU-2. On neutralization effect, PNGS mutations at N197 (C2), N301 (V3), N442 (C4) and N625 (gp41) rendered the virus more susceptible to neutralization by the monoclonal antibodies (MAbs) that recognize the CD4 binding site or gp41. Generally, mutations on V4/V5 loops, C2/C3/C4 regions and gp41 reduced the neutralization sensitivity to PG16. However, mutation of N289 (C2) made the virus more sensitive to both PG9 and PG16. Furthermore, we showed that mutations at N142 (V1), N355 (C3) and N463 (V5) conferred resistance to neutralization by anti-gp41 MAbs. We used the available structural information of HIV Env and homology modeling to provide a structural basis for the observed biological effects of these mutations.

**Conclusions:**

This report provides the first systematic experimental account of the biological role of the entire PNGS on an HIV-1 Env, which should provide valuable insights for understanding the function of Env in HIV infection cycle and for developing future anti-HIV strategies.

## Background

The human immunodeficiency virus type 1 (HIV-1) Env consists of a trimer of heterodimers of the gp120 surface protein and the gp41 transmembrane protein [1-3]. HIV-1 gp120 is responsible for binding both the target cell CD4 receptor and co-receptors (CCR5 or CXCR4) [4,5], while gp41, together with gp120, mediates fusion of the viral and host cell membranes for cell entry [6]. The gp120 monomer has five highly conserved (C1-C5) regions and five variable (V1-V5) regions. Crystal structures of gp120 reveal that these regions can be organized into four structural domains: the inner and outer domains, a 4-stranded bridging sheet, and the V1/V2 domain that has been determined recently [7,8]. Upon CD4 receptor binding, gp120 inner domain undergoes major structural rearrangements to allow for bridging sheet formation; whereas the majority of the outer domain appears to remain essentially unchanged [9]. Subsequent co-receptor binding by areas located on the bridging sheet and V3 loop from the outer domain triggers additional gp120 conformational changes that promote eventual gp120 dissociation from gp41 and transition of gp41 into different structural forms that are necessary for viral-host membrane fusion [10,11].

This cascade of conformational changes leads to the exposure of new epitopes on gp120 and gp41 for antibodies to recognize. Classes of broadly neutralizing monoclonal antibodies (MAbs) have been shown to neutralize HIV-1 by binding different regions of Env, including the gp120 CD4 binding site (b12, VRC01, VRC03), the membrane proximal region of the gp41 ectodomain (2F5, 4E10), and clusters of glycans on the surfaces of gp120 (2G12, PG9, PG16) [12]. However, owing to the steric and kinetic constraints caused by the continual structural rearrangements that occur, some of these epitopes are only transiently exposed.

HIV-1 gp120 is heavily glycosylated by the infected host with glycan moiety comprising about 50% of its total mass [13]. These glycans influence Env conformations/oligomerization, and affect viral entry, infectivity and antibody recognition [8,14-16]. Indeed, N-linked glycans are essential for correct folding and processing of gp120 and for the structural rearrangements of gp120 that occur during CD4 and co-receptor binding that mediate membrane fusion and cell entry of HIV-1 [17]. Additionally, the dense glycans on the outer domain protect the virus from antibody-mediated neutralization [18]. In gp41 of most HIV-1 isolates, there are four consensus N-linked glycosylation sites within a region flanked by two highly conserved vicinal cysteines and a hydrophobic membrane anchor domain [19]; however, little is known about the function of the N-linked glycans on gp41. Despite the vast literature on the N-linked glycosylation of gp120 and gp41, the impact of individual N-linked glycans on HIV-1 infectivity and antibody-mediated neutralization has not been systemically evaluated before.

The circulating recombinant forms (CRFs) of HIV-1, CRF07_BC and CRF08_BC, are descendants of the parental subtypes B from Thailand and C from India, and are comprised of mostly subtype C in *envelope*. The CRF07_BC recombinant strain has been one of the most predominantly circulated HIV-1 strains in China. The total potential N-linked glycosylation sites (PNGS) on CRF07_BC Env range between 24–35 (mean=30), with a mean of 25.8 in gp120 (range 20–30) and 4.2 in gp41 (range 2–6) [20]. The wild-type (wt) envelope, FE, was cloned in our laboratory from a HIV-1 subtype 07_BC infected patient obtained in Guangxi province in China. 25 PNGS exist on FE Env, 21 in gp120 and 4 in gp41.

In the present study, the effect of PNGS on viral infectivity and antibody-mediated neutralization was evaluated through systematic mutations of each PNGS of Env and using pseudoviruses expressing the mutated Env to assess the infectivity and sensitivity to the MAb-mediated neutralization. In addition, we have utilized structural data of gp120 and gp41 and molecular modeling to evaluate the structural/functional relationship of the PNGS based on what we have observed in our systematic mutational study.

## Results

### Analysis of FE PNGS conservation among HIV-1 Isolates

We aligned the FE Env sequence with other HIV-1 isolates (12 isolates of each clade): clade B (JRCSF, JR-FL, SF162, YU-2, NL4-3, 89.6 and B01-06 [21]); clade BC (FE, sc19-15, hb5-3, sc21-28, xj74-2, xj180-29, bj23-1, sc20-15, sc22-16, yn99r-5, yn148r-9 and yn177-1 [20]); clade AE (GX91.2, GX81.43, GX24.8, GX71.18, GX28.31, GX88.47, GX90.1, BJX4.6, GX74.20, GX34.21, GX35.33 and BJ5.11 [22]) to analyze the PNGS conservation. 20 out of the 25 PNGL sites in FE strain are present in all or most strains (Table 1), suggesting their high degree of conservation. The 3 PNGS only present in subtype BC Env (underlined in Table 1) are all located on V1/V2/C4. Generally speaking, the PNGS in the constant regions (except N442) and gp41 are highly conserved (Table 1).

**Table 1:** Conservation of all 25 PNGS sites in FE among 36 HIV-1 strains

**PNGS**	**Region**	**HIV-1**		
		**BC (% Cons.)**	**B (% Cons.)**	**AE (% Cons.)**
**N88**	**C1**	**100**	**100**	**100**
**N133**	**V1**	**50**	**8**	**8**
**N142**	**V1**	**75**	**0**	**0**
**N156**	**V1**	**100**	**100**	**100**
**N160**	**V1**	**92**	**75**	**92**
**N181**	**V2**	**92**	**0**	**0**
**N197**	**C2**	**100**	**92**	**100**
**N234**	**C2**	**92**	**58**	**92**
**N241**	**C2**	**100**	**100**	**100**
**N262**	**C2**	**100**	**100**	**100**
**N289**	**C2**	**100**	**83**	**100**
**N301**	**V3**	**100**	**100**	**92**
**N339**	**C3**	**100**	**83**	**8**
**N355**	**C3**	**92**	**100**	**0**
**N392**	**V4**	**100**	**75**	**67**
**N408**	**V4**	**25**	**42**	**0**
**N411**	**V4**	**50**	**75**	**0**
**N442**	**C4**	**67**	**0**	**0**
**N448**	**C4**	**100**	**100**	**100**
**N463**	**V5**	**33**	**42**	**8**
**N466**	**V5**	**50**	**42**	**42**
**N611**	**gp41**	**100**	**100**	**100**
**N616**	**gp41**	**100**	**92**	**100**
**N625**	**gp41**	**100**	**100**	**100**
**N637**	**gp41**	**100**	**100**	**92**

### Effect of PNGS Mutations on viral infectivity

**Figure 1:**
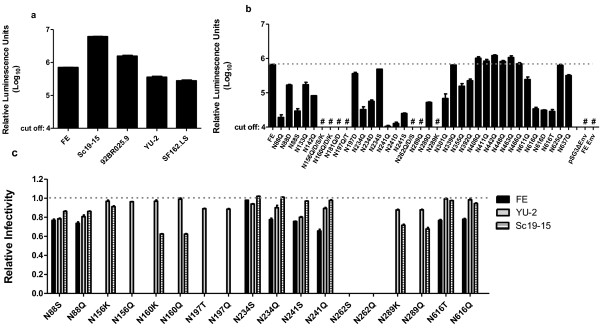
**Infectivity assay of wt HIV strains and the PNGS mutants. a**) Comparison of the infectivity of wt HIV-1 strains: FE and four other strains. **b**) Infectivity assay result of all FE PNGS mutants. **c**) Comparison of relative infectivity of some of the PNGS mutants in FE, Sc19-15 and YU-2 isolates. Data are shown as relative luminescence units (RLU) in a logarithmic scale. Relative infectivity was calculated by dividing the Log_10_ (RLU of mutant) by Log_10_ (RLU of wt). The data represent the means of three independent experiments, and the error bars indicate the standard deviations from the means. The cut off value of the infectivity assay is 4.0. #, glycan deletion mutant that could not support virus entry.

We compared the infectivity of FE with that of YU-2 (clade B), SF162.LS(clade B), 92BR025.9 (clade C) and sc19-15 (clade 07_BC), and found that the infectivity of FE strain was similar to those of the other HIV-1 strains from the different clades (Figure 1a).

**Figure 2:**
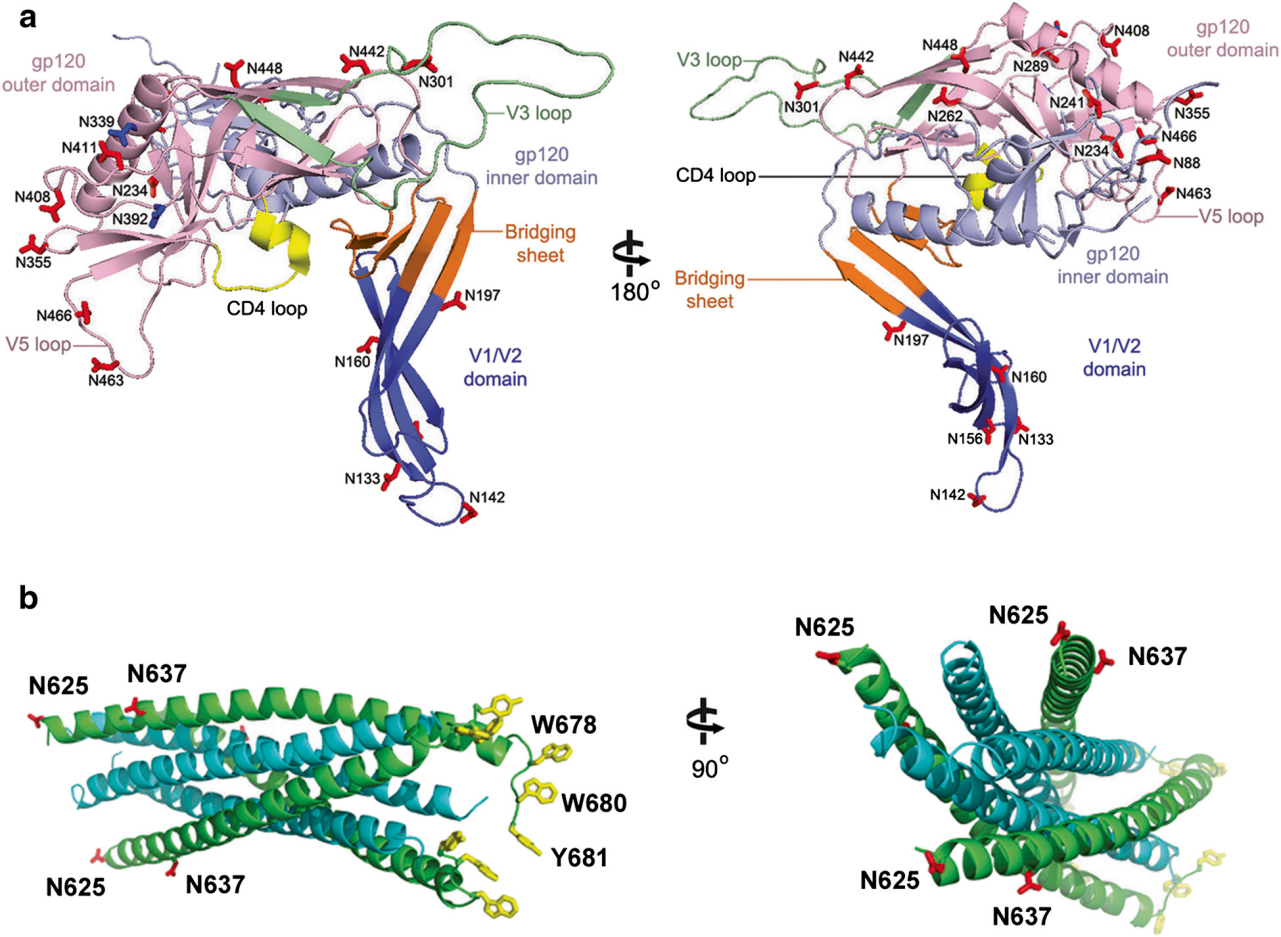
**Structure models of FE Env, gp120 and gp41. a**) Two views of the model of the FE gp120 monomer with grafted V1/V2 domain (see Methods). The gp120 monomer consists of four domains: the inner domain (light blue), the outer domain (light pink), the bridging sheet (orange), and the V1/V2 loop (dark blue). The 4 beta stranded bridging sheet forms upon CD4 binding near the CD4 binding loop (yellow). The V3 loop (light green) extends from the outer domain. Mutated PNGS that increase or decrease viral neutralization by any of the MAbs are shown as red sticks, and sites with negligible effects are shown as blue sticks. **b**) Two views of the model of the FE gp41 six helix bundle (see Methods). The three alpha helices in the middle are shown in cyan, and the three helices on the outside are in green. Mutated PNGS N625 and N637 are shown as red sticks. Aromatic residues W678, W680, and Y681 (yellow) from the MPRE region extend from the tip of each monomer and are proposed to insert into the viral membrane (only one set of residues shown).

In order to determine whether Env mutants with PNGS mutations could be incorporated into viral particles and support viral infection, we generated pseudoviruses with the PNGS mutants of Env and tested the capacity of these pseudoviruses to infect TZM-bl cells (CD4^+^, CCR5^+^, and CXCR4^+^). Except for N181, the location of all gp120 PNGS can be visualized on the FE gp120 monomer model as shown in Figure 2a. All of the PNGS except for N392 are predicted to be on the surface of both the inner and outer domains and the V1/V2 domain (Figure 2a). Two of the gp41 mutations, N625 and N637, can be modeled using available crystal structures of the gp41 core six helical bundle, which is proposed to be inserted into both the cellular and viral membranes for membrane fusion (Figure 2b) [23]. N625 is located on the linker region between the Heptad Repeat 1 (HR1) and HR2 alpha helices, and N637, located on HR2, is predicted to be on the outer surface of the six helical core (Figure 2b). Residues N611 and N616 are predicted to be on a loop in the linker region between HR1 and HR2 and are disordered in the structures, and therefore, cannot be visualized in the model.

Among these 25 mutants with N->Q mutations individually, nine (N88Q, N156Q, N160Q, N181Q, N197Q, N234Q, N241Q, N262Q, N289Q and N616Q) had significant reduction in viral infectivity (Figure 1b) (*p*<0.05). Six of these ten mutants (N156Q, N160Q, N181Q, N197Q, N262Q and N289Q) were non-infectious. These nine PNGS were mainly located in the C1, V1/V2, C2 regions and one in gp41 (N616Q). Another group of seven mutants (N133Q, N142Q, N301Q, N355Q, N392Q, N611Q and N637Q) showed slight reduction of viral infectivity (*p*<0.05). There are five mutants (N408Q (V4), N411Q (V4), N442Q (C4), N448Q (C4) and N463Q (V5)) that had a significant increase in viral infectivity (*p*<0.05), and three mutants (N339Q, N466Q and N625Q) showed no difference in viral infectivity compared to the wt.

To determine the effects of substitution other than Gln (Q) at PNGS, we mutated to Asp (D) (N->D) for the PNGS at positions 88, 156, 160, 181, 197, 234, 241, 262, 289 and 616. Interestingly, compared with the completely inactive N197Q and N289Q mutants, N197D and N289D recovered some level of viral infectivity (Figure 1b, *p*>0.05). Additionally, N88D showed a ~9-fold increase in infectivity compared to N88Q mutant and N234D showed a ~2-fold increase. Others showed no difference in viral infectivity compared with the N to Q mutants.

For these PNGS (N156, N160, N181, N197, N262 and N289) with N->Q mutation that resulted in complete loss of the infectivity, we also made the S/T to A mutation to abrogate the glycosylation motif (NXS/T, X can be any amino acid except proline). We found that the S/T->A mutants showed no difference in viral infectivity compared to the N->Q mutants, and that no activity was detected in both N->Q and S/T->A mutants (data not shown). These data suggest that the loss of infectivity of N->Q mutation in these six mutants probably is due to the loss of glycosylation.

The effect of PNGS mutations on viral infectivity in different HIV-1 isolates was also investigated by generating PNGS mutations in other isolates that belongs to different clades. We made mutations in HIV isolates Sc19-15 (clade BC) and YU-2 (clade B) on those PNGS sites that significantly influenced the FE infectivity (N88, N156, N160, N197, N234, N241, N262, N289 and N616) by performing both N->Q and N->K/T/S mutations. Additionally, we also made N->K/T/S mutations on these sites in FE isolates. The result showed that N->K/T/S/Q mutation of N156, N160, N197, N262 and N289 resulted in complete loss of the infectivity for FE, most of these mutations (except for N156 and N289) also lost infectivity for the Sc19-15 isolate (Figure 1c). Similarly, the mutational effect of N->Q mutation of these PNGS sites was largely the same between FE and Sc19-15. However, among the mutations in YU-2, only one site, N262->Q/S showed complete loss of infectivity (Figure 1c). For those mutations that still retained relatively high amount of infectivity in FE, YU-2 and Sc19-15 isolates, all PNGS mutations (except N234S) showed a slightly reduced infectivity (but consistent) in FE than in the other two isolates.

### Effect of the PNGS Mutations on gp120 incorporation into virions

**Figure 3:**
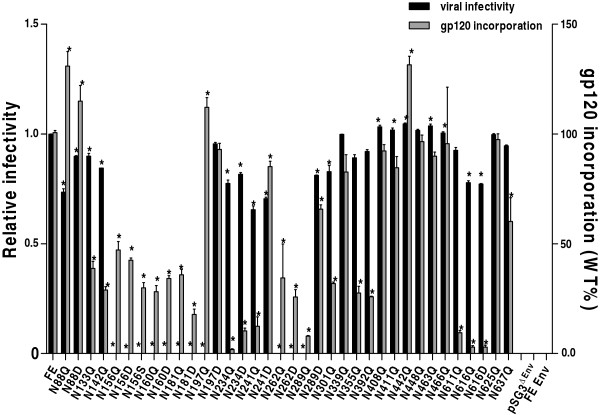
**Viral relative infectivity and virion incorporation of mutant gp120.** Gp120 incorporation data are shown as the percentage of the gp120/p24 ratio of the wt FE pseudovirus, with the wt gp120 incorporation set to 1.0. Relative infectivity was calculated by dividing the Log_10_ (RLU of mutant) by Log_10_ (RLU of wt). The data represent the means of three independent experiments, and the error bars indicate the standard deviations from the means. *P* values were calculated by using One-way analysis of variance (Tukey’s multiple comparison Test, SPSS 10.0). The difference of the mean infectivity and gp120 incorporation between wt and mutant is considered to be significant when a *p* value is <0.05, which is indicated with an asterisk in the figure.

To establish whether the loss of infectivity is caused by solely the mutations of PNGS or by a defect in the mutant gp120 incorporation into virions, we measured virus-associated gp120 glycoprotein for each PNGS mutant of FE Env. The mutant viruses were pelleted through a 25% sucrose cushion, and then subjected to p24 and gp120 ELISAs. The ratios of gp120/p24 were calculated for each virus to measure gp120 incorporation into virions, and are shown in Figure 3 as the percentage of wt. In the control experiments, cells were transfected with only plasmid pSG3^ΔEnv^ alone (expressing p24) or FE Env-expressing plasmid alone (expressing gp120). As expected, after ultra-centrifugation, no gp120 was present, but a similar level of p24 was detected from the single pSG3^ΔEnv^ transfected pellet, and neither p24 nor gp120 were detected from the FE Env-expressing plasmid transfected pellet.

Among all Env mutants, N88Q and N442Q in-creased ~30% of gp120 incorporation compared to wt (*p*<0.05) (Figure 3). N88D and N197Q showed slightly enhanced gp120 incorporation (~12% of wt, *p*>0.05). Nine mutants (N197D, N241D, N339Q, N408Q, N411Q, N448Q, N463Q, N466Q and N625Q) had slightly reduced gp120 incorporation than wt (around 83% to 97% of wt), mutants N289D and N637Q showed a 60%-66% of wt level. Much lower levels of virus-associated gp120 glycoprotein (2% - 47% of wt) was observed for mutants N133Q, N142Q, N156Q/D/S, N160Q/D, N181Q/D, N234Q/D, N241Q, N262Q/D, N289Q, N301Q, N355Q, N392Q, N611Q, N616Q/D (*p*<0.05).

While the gp120 incorporation is largely the same for the N->Q mutants and N->D mutants on the same PNGS, we found that N->D mutation at position N241 and N289 has much higher gp120 incorporation than the N->Q mutations (Figure 3, *p*<0.05). The gp120 incorporation for N241Q was 12% of wt, but for N241D was 85% of wt, even though both mutants essentially lost all infectivity (Figure 3). However, for N289Q to N289D, gp120 incorporation was 8% to 66% of wt, respectively, which may correlate with the increase of infectivity from undetectable for N289Q to 81% of wt for N289D (Figure 3).

### Effect of PNGS Mutations on Neutralization by MAbs

In the present study, 18 individual PNGS mutants were tested in order to characterize their neutralization phenotype by a panel of MAbs: b12, 2F5, 4E10, VRC01, VRC03, PG9 and PG16. All other mutants that showed significant reduction of viral infectivity or pseudovirus formation were excluded for the neutralization study.

**Figure 4:**
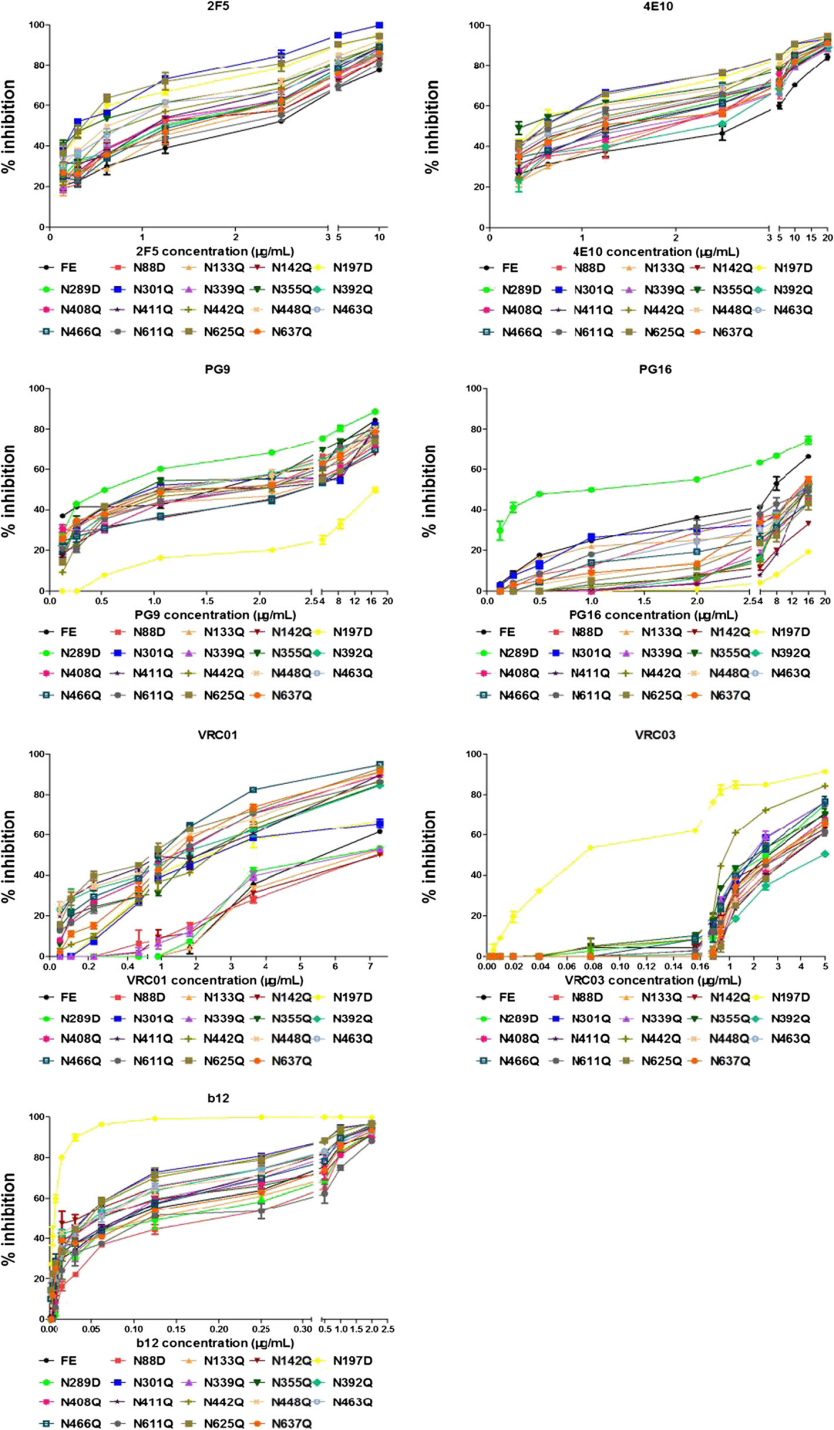
**Neutralization susceptibility of WT FE and PNGS mutants by several known neutralizing MAbs.** All data points represent the means and the error bars indicate the standard deviations from the means that were obtained from three independent experiments. Neutralization assays of the pseudovirions produced from WT and mutants were described in Materials and Methods.

As shown in Table 2 and Figure 4, different PNGS mutations displayed varied sensitivity to different MAbs. Among these mutants, the N197D showed a most profound effect on the increase of susceptibility for a number of MAbs. This N197D displayed a 37-fold increase in susceptibility to neutralization by VRC03, 17-fold increase to MAb b12, 5-fold to both 2F5 and 4E10 (~5-fold), and 2-fold increase to VRC01.

**Table 2:** Neutralization of Env glycosylation mutants by MAbs

**Env**	**Region**	**MAbs/IC**_**50**_**(μg/mL)(%)**							
		**Percentage of neutralization sensitivity relative to WT**							
		**2F5**	**4E10**	**b12**	**VRC03**	**VRC01**	**PG9**	**PG16**	**3869**
**FE(WT)**	**-**	2.224 (100)	2.986 (100)	0.088 (100)	2.601 (100)	5.500 (100)	1.415 (100)	7.600 (100)	>25 (100)
**N88D**	**C1**	1.097 (203)	1.840 (162)	0.201 (44)	3.427 (76)	4.936 (111)	0.988 (143)	12.847 (59)	ND
**N133Q**	**V1/V2**	1.571 (142)	1.776 (168)	0.123 (72)	2.962 (88)	5.178 (106)	2.479 (57)	16.233 (47)	5.035 (>496)
**N142Q**	**V1/V2**	1.117 (199)	0.999 (299)	0.035 (251)	2.944 (88)	5.100 (108)	3.041 (47)	16.350 (46)	1.315 (>1901)
**N197D**	**V1/V2**	0.396 (562)	0.477 (626)	0.005 (1760)	0.070 (3716)	2.355 (234)	16.123 (9)	>25 (<30)	5.540 (>451)
**N289D**	**C2**	1.250 (178)	1.544 (193)	0.150 (59)	2.539 (102)	3.800 (145)	0.519 (273)	0.731 (1040)	ND
**N301Q**	**V3**	0.210 (1059)	0.585 (510)	0.040 (220)	1.235 (211)	2.330 (236)	0.875 (162)	14.884 (51)	ND
**N339Q**	**C3**	1.127 (197)	1.579 (189)	0.083 (106)	1.799 (145)	4.899 (112)	1.664 (85)	14.644 (52)	ND
**N355Q**	**C3**	0.426 (522)	0.353 (846)	0.060 (147)	1.980 (131)	5.456 (101)	0.800 (177)	15.250 (50)	ND
**N392Q**	**V4**	1.265 (176)	2.327 (128)	0.051 (173)	4.908 (53)	5.678 (97)	1.091 (130)	14.064 (54)	ND
**N408Q**	**V4**	1.505 (148)	2.010 (149)	0.063 (140)	2.938 (89)	5.777 (95)	1.775 (80)	16.512 (46)	ND
**N411Q**	**V4**	1.072 (207)	1.302 (229)	0.082 (107)	3.511 (74)	5.230 (105)	0.900 (157)	14.783 (51)	ND
**N442Q**	**C4**	0.781 (285)	0.934 (320)	0.045 (196)	0.791 (329)	4.120 (133)	1.650 (86)	16.111 (47)	ND
**N448Q**	**C4**	0.635 (350)	0.694 (430)	0.057 (154)	2.917 (89)	4.987 (110)	0.878 (161)	13.386 (57)	ND
**N463Q**	**V5**	0.763 (291)	0.895 (334)	0.056 (157)	2.170 (120)	3.450 (159)	2.544 (56)	16.333 (47)	ND
**N466Q**	**V5**	1.339 (166)	1.397 (214)	0.087 (101)	2.139 (122)	3.204 (172)	3.070 (46)	16.378 (46)	ND
**N611Q**	**gp41**	1.836 (121)	0.704 (424)	0.116 (76)	3.103 (84)	5.431 (101)	1.797 (79)	15.830 (48)	ND
**N625Q**	**gp41**	0.400 (556)	0.577 (518)	0.041 (215)	3.329 (78)	5.398 (102)	1.069 (132)	16.450 (46)	ND
**N637Q**	**gp41**	1.429 (156)	1.168 (256)	0.102 (86)	2.812 (92)	5.178 (106)	1.342 (105)	13.411 (57)	ND

Percentage of neutralization sensitivity relative to WT (%) = IC_50_ WT **/** IC_50_ mutant.

*ND*: not detected.

A few other mutants also showed a modest, but reproducible increase of susceptibility to some MAbs. For example, N301Q showed an approximately 10-fold increase in susceptibility to neutralization by 2F5, a 5-fold increase to 4E10, and a 2-fold increase to b12, VRC01 and VRC03 (Table 2, Figure 4). N142Q showed about a 2-fold increase in susceptibility to 2F5, 4E10 and b12. N289D showed a 10-fold increase in susceptibility PG16 and a 2-fold increase to PG9. N355Q showed a ~5-fold increase in susceptibility to 2F5 and a ~8-fold increase in susceptibility to 4E10.

A few of the PNGS mutants also displayed reduced susceptibility to MAbs PG9 and PG16 (Table 2, Figure 4). N197D had an 11-fold reduced susceptibility to neutralization by PG9, and N142Q had a ~2-fold reduced susceptibility to neutralization to PG9. About half of the PNGS mutants also showed around 2-fold reduction in susceptibility to PG16.

The FE and all three mutants on V/V2 (N133Q, N142Q and N197D) were tested with four different monoclonal antibodies targeting areas of the V3 domain: 447-52D, 2191, 2442 and 3869. All four strains were resistant to mAbs 447-52D, 2191 and 2442 at highest IC_50_ (25 μg/mL). However, even though wt FE also was resistant to mAb 3869, all of the three V1/V2 mutants of FE became more sensitive to mAb 3869, with the N142Q mutant being 19 fold more sensitive (Table 2). This result suggests that V1V2 may shield part of the V3 domain on an adjacent monomer within an assembled trimer.

## Discussion

We made a complete set of systematically mutated N-glycosylation Env mutants and conducted comprehensive evaluation and analysis on their effects on viral infectivity and neutralization by different classes of neutralizing MAbs.

### PNGS mutations and viral infectivity

**Figure 5:**
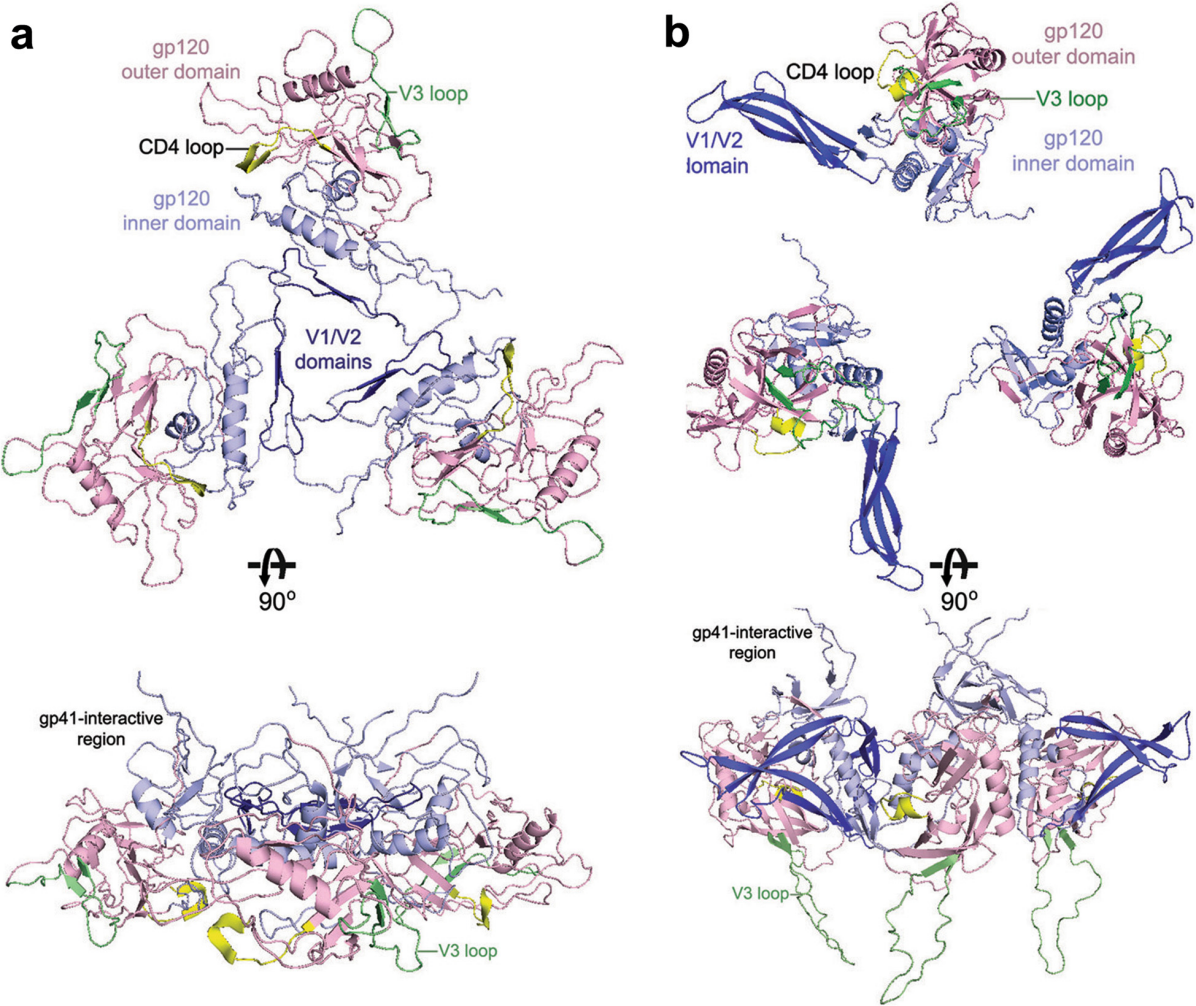
**Modeled FE gp120 trimers. a**) Model of unliganded FE gp120 trimer containing the truncated V1/V2 stem loop in a “closed” conformation (Upper and lower panels). Top and front views showing the closed conformation of an HIV-1 FE gp120 trimer. We were unable to model the recently solved complete V1/V2 domain onto the unliganded trimer model due to steric clashes; however, a portion of the truncated V1/V2 domain, the V1/V2 stem loop (dark blue), can be seen in a “closed” conformation near the center of the trimer. More structural information is needed regarding the unliganded full-length gp120 trimer to determine precisely where the complete V1/V2 domain is positioned in this conformation; however, owing to its flexibility, the V1/V2 domain will likely also be positioned near the center of the trimer. This is also consistent with cryo-electron tomography studies [26,28]. **b**) Model of CD4 bound FE gp120 trimer containing the complete V1/V2 domain in an “open” conformation. Top and front views showing the open conformation of the trimer upon binding the CD4 receptor. We were able to graft the complete V1/V2 domain (dark blue) onto the gp120 core. Upon CD4 binding, the V1/V2 domain rotates outwardly in an “open” orientation, consistent with cryo-electron tomography studies [26,28].

We showed that all Env mutations on or near the V1/V2 domain had no infectivity or a reduced viral infectivity (see Figure 3: N156Q/D/S, N160Q/D, 181Q/D and N197Q inactive; N133Q, N142Q, N197D reduced activity). Previous studies suggest that the V1/V2 domain undergoes CD4-induced conformational changes to exposure the co-receptor binding site [24]. A recent co-crystal structure of V1/V2-PG9 shows that the V1/V2 orientation varies dramatically in the context of the monomeric gp120 [8]. To gain structural insights of the glycan mutants located on V1/V2, we grafted a model of the FE V1/V2 domain onto the FE gp120 core model (Figure 2a). We also analyzed how this model may oligomerize in a gp120 trimer based on models from cryo-ET [25]. Even though modeling the V1/V2 domain based on the unliganded trimer model had steric clashes (Figure 5a), no clashes are present when modeling is based on the gp120 trimer model in the CD4-bound form (Figure 5b). Our model is consistent with cryo-ET studies suggesting that the V1/V2 domain undergoes repositioning in the “closed” and “open” conformations of the Env trimer [25,26]. This model also agrees with studies that propose that the V1/V2 domain from one monomer may interact with an adjacent monomer in the trimer to shield the neighboring V3 loop from antibody binding [27]. Our neutralization test of infectivity of three V1/V2 mutants of FE using anti-V3 mAb 3869 showed a marked increase of sensitivity, providing evidence supporting such a shielding hypothesis.

The long V3 loop extends from the gp120 outer domain that remains fairly stable during receptor/co-receptor binding; thus, it is not thought to undergo large conformational changes as V1/V2 domain [29]. This glycosylated loop determines the specificity of the co-receptor and could act as a “molecular hook” that binds the co-receptor and perhaps other gp120 molecules in the trimer (Figure 2a and 5a, lower panel) [29,30]. Thus, N301 at the stem of the V3-loop may play a role in stabilizing the V3 and modulating co-receptor binding (Figure 2a). Therefore, the drastic decrease of viral infectivity seen by mutant N301Q may be due to a decrease in co-receptor binding. Interestingly, N442Q showed almost a 2-fold increase in viral infectivity (Figure 3). This residue is located on a loop neighboring the V3-loop and N301 (Figure 2a). Thus, removal of the glycan may indirectly increase viral activity by causing a shift in the V3 loop, leading to an increase in co-receptor binding.

PNGS mutants N262Q/D and N289Q showed a complete lack of viral infectivity (Figure 3). Interestingly, N289D displayed viral infectivity (Figure 3), and showed much higher gp120 incorporation than N289Q. We could not offer a good explanation for this observation. These residues are located right in the junction between the inner and outer domains of gp120 that undergoes major conformational changes (Figure 2a) [7,31], thus, these mutations could affect the conformational changes that are necessary for cell entry. Previous studies also indicate the importance of glycosylation at N262 for gp120/gp41 expression in the viral particle [32]. Thus, glycans in this area are likely important for proper gp120 folding and structural rearrangements.

Although there is no available co-crystal structure of gp120 with gp41, studies indicate that residues on the gp120 inner domain β-sandwich are important for gp41 interaction and gp41 transitions [33]. Residues N88, N234, and N241 in this region have previously been identified in the non-covalent gp120-gp41 interactions (Figure 2a and 5a, lower panel “gp41 interactive region”) [34-39]. Therefore, reduced viral infectivity of these mutations may be a result of inefficient viral entry due to lack of proper interactions of gp120 with gp41.

CD4 and co-receptor binding initiate structural changes in gp120 and gp41 that mediate fusion of the viral and cellular membranes. Three gp41 states have been proposed: the prefusion, the prehairpin intermediate, and the post-fusion conformations [10]. We generated a FE gp41 model based on the gp41 late fusion intermediate state, which is thought to stabilize membrane fusion pore opening (Figure 2b) [23]. The model is a 6-helix bundle core (Figure 2b). Our studies show that mutation of gp41 residues 611, 616, and 637 reduced viral infectivity, whereas N625Q showed no change in viral infectivity (Figure 3). N625 and N637 can both be visualized on our FE gp41 model (Figure 2b) [23]. However, N611 and N616, although present in the crystalized gp41 constructs, are not visible due to their location on the flexible loop. Previous studies showed that only one of the glycans at position 611 and 616 is actually glycosylated, likely only N611 [40,41]. In our FE gp41 model, N637 is located on the outer surface of the helix bundle (Figure 2b), which is consistent with the previous report that N637 is glycosylated [40,41]. Nonetheless, mutation at N637 only had a relatively small effect on the infectivity (Figure 3). N611 and N616, located on the linker region, may play a role in other gp41 conformations that could affect gp120 incorporation into virion (Figure 3), and mutations of these residues resulted in more loss of infectivity. N625 points away from the helix bundle (Figure 2b), which would explain why its mutation does not affect viral infectivity.

A previous study, which focused on the effect of 28 PNGS in viral infectivity of a clade B virus, reported that only three sites (N262, N332 and N386) are detrimental for infectivity for isolate YU-2 and that only N262 is also detrimental in another clade B isolate JRFL [42]. In our study, we also showed that the same N262 is detrimental for infectivity for clade BC (isolates FE and Sc19-15) as well as for clade B (isolates YU-2 and NL4-3 [32]). However, we found that N332 and N386 were dispensable for infectivity for the clade BC virus. In addition, we found that mutation of six other PNGS mutants (N156Q, N160Q, N181Q, N197Q, N262Q and N289Q) in the FE isolate resulted in non-infectious virus and mutation of most of these sites yielded similar results in another clade BC isolate Sc19-15. Our data would imply that there are dramatic differences in the glycan dependence of clade B and clade BC isolates. Clade B appears to be insensitive to most glycan removals, with only 1–3 sites being important for infectivity, while the infectivity of clade BC isolates are much more sensitive to the mutation of many more glycan sites.

### PNGS mutations and MAb neutralization

For those glycosylation mutants that showed viral infectivity, we examined their sensitivity to seven broadly neutralizing MAbs [43,44]. The neutralization ability of CD4bs MAbs showed a marked increase for the N197D mutant, with b12 displaying a >17-fold increase over wt FE and VRC03 also displaying a >37-fold increase (Table 2). Studies show that the N197 mutation significantly increases b12 susceptibility of HIV strains JR-CSF, 89.6, and CRF01_AE [45-47]. As previously discussed, N197 is located near the stem of V1/V2 that undergoes conformational changes and shields the bridging-sheet, which may affect CD4 receptor and co-receptor binding. Therefore, removal of the N197 glycan could result in the removal of the glycan shield, and/or a shift of V1/V2, which may allow the CD4bs MAbs to better access their epitopes, thus increasing sensitivity to neutralization. Interestingly, the structures of the gp120 core (missing V1/V2) in complex with the three CD4bs-MAbs show that they are positioned differently, with b12 being the closest, VRC03 next, and VRC01 the farthest to V1/V2 [48-50]. This positioning relative to the V1/V2 domain may explain why b12 and VRC03 show a higher increase in neutralization by the N197 mutant compared with VRC01. It is also possible that the V1/V2 conformational changes could affect adjacent binding sites in the trimer [47]. Our data also indicate that the V1V2 region of one monomer may shield part of the V3 binding site on an adjacent monomer within a trimer.

Unlike our results seen from the CD4bs MAbs, almost all of the PNGS mutants tested displayed a 2–10 fold increase in susceptibility to neutralization by the anti-gp41 MAbs, 2F5 and 4E10 (Table 2). Both 2F5 and 4E10 act to inhibit the fusion process by binding the highly conserved Membrane Proximal External Region (MPER) region of gp41 [51,52]. The MPER region of gp41 may only be exposed during the transient prehairpin intermediate state in which gp41 is in an extended conformation [10]. Hence, 2F5/4E10 would only have a short time period to bind their epitopes, which may explain why the 2F5- and 4E10-like antibodies are rarely found in HIV-1-infected plasma [10]. Any conformation changes in gp120/gp41 that would enhance receptor binding may promote increased neutralization by 2F5/4E10. In our study, 2F5 and 4E10 showed an increase in neutralization to the majority of our mutants, indicating that the presence of glycans may somehow interfere with anti-gp41 MAb binding (Table 2). Similar to our results, deglycosylation of Env enhanced binding of 2F5 and 4E10 MAbs [53]. These results may indicate that removal of glycans may allow more flexibility for gp120/gp41 rearrangement that leads to the exposure of the MPER region of gp41.

Quaternary-specific MAbs PG9 and PG16 recognize gp120 epitopes composed of glycans on the V1/V2 domain [54]. A recent crystal structure shows that two glycans (N156/N160) and a V1/V2 β-strand form the PG9 recognition site [8]. One conspicuous observation in this study is that PG9 and PG16 are the only MAbs that displayed decreased neutralization activity for some PNGS mutants, with PG16 having a more dramatic decrease (higher IC_50_) than PG9 (Table 2). Furthermore, PG9/PG16 MAbs show major decreases in neutralization toward the N197D mutant. Because N197 is near the V1/V2 domain, this glycan may also affect the PG9/P16 epitope. Alternatively, perhaps N197D affects the positioning of the V1/V2 domain within the Env trimer. Based on our model of the FE trimer in unbound and CD4-bound form, the V1/V2 domain undergoes major conformational changes (Figure 2b and 5a). In the CD4-unbound “closed” state, the gp120 monomers and their V1/V2 domains are packed tightly together at the apex (Figure 5a), whereas, after CD4 binding, the V1/V2 rotate outward in the “open” state (Figure 5b) [25]. As PG16 recognizes the gp120 epitope significantly better in trimer form, perhaps the N197D mutant alters the conformation of the V1/V2 domain in the trimer resulting in a shift of the epitope to reduce PG16 binding.

Similar to PG9/PG16, MAbs PGT127 and PGT128 are also glycan dependent for neutralizing activities. PGT128 recognizes two conserved glycans at N301/N332 as well as a short β-strand segment of the gp120 V3 loop [55]. Substitutions at N332/N301 resulted in the loss of neutralizing activity against most isolates. Previous analysis revealed that N295, N332, N339, N386, N392 and N448 are likely involved in the 2G12 epitope [56].

In the present study, our results indicated that wt and all PNGS mutants of FE were resistant to 2G12 at the highest concentration tested (25 μg/mL MAbs) (data not shown). FE lost the N295, N332 and N386 PNGS that were suggested to be involved in 2G12 binding. We re-introduced these three PNGS into FE Env together, which remained resistant to 2G12 in our experiments (data not shown). 2G12 broadly neutralizes clade B isolates and is less effective against other clades, particularly clade C viruses, which have a somewhat different oligomannose glycan arrangement than clade B viruses. The continued insensitivity to 2G12 for the re-introduced mutant suggests the role of other residues in forming 2G12 epitope. A previous study showed that 447-52D can neutralize most subtype B viruses which contain the peptide GPGR at the tip of the V3 tip, but infrequently neutralized other clades [57]. The wt FE and all mutants are all GPGQ at the tip of the V3 loop, this may explain why the viruses in our study are resistant to mAb 447-52D.

## Conclusions

In summary, through systematic mutations of all the PNGS of the Env gp120 and gp41 proteins, we have charted for the first time the relative significance of each of the PNGS of the FE Env for their biological functions in virion formation, viral infectivity, and MAb sensitivity. Using molecular modeling based on the available rich structural information in the literature, we provide the structural and functional correlation for the biological effects of most of the PNGS observed in our study, which should be valuable for our understanding of the roles of the respective glycans in HIV infection and host immunity. Furthermore, the results of the increased or decreased sensitivity to neutralizing antibodies after PNGS mutations will be valuable information for future design of anti-HIV/AIDS strategy.

## Methods

### Cells and plasmid

TZM-bl (JC53-bl) cells, Env-deficient HIV-1 backbone (pSG3^ΔEnv^) were obtained from the US National Institutes of Health (NIH) AIDS Research and Reference Reagent Program (ARRRP), as contributed by John C. Kappes, Xiaoyun Wu, and Tranzyme (Birmingham, AL). The 293FT cells were obtained from Invitrogen (Carlsbad, CA).

### MAbs

MAbs 2F5, 4E10, 2G12 and b12 were obtained from the International AIDS Vaccine Initiative (IAVI, New York, NY); MAbs 2F5 and 4E10 were contributed by Hermann Katinger (Institute of Applied Microbiology, Vienna, Austria) and b12 was contributed by Dennis Burton and Carlos Barbas (The Scripps Research Institute, La Jolla, CA); MAbs VRC01, VRC03, PG9, PG16 and anti-V3 MAbs (447-52D, 2191, 2442 and 3869) were obtained from the ARRRP (NIH).

### Elimination of PNGS by mutagenesis

The motif for an N-linked glycosylation site is Asn-X-Thr/Ser, where X can be any amino acid except proline [58]. Elimination of PNGS was performed using site-directed mutagenesis, by changing an asparagine (N) to a glutamine (Q) or aspartate (D). The wt Env gene was inserted into pcDNA 3.1D/V5-His-TOPO (Invitrogen) as a template for mutagenesis. Mutagenesis was performed using approximately 50 ng plasmid DNA template and 1 μL of 1 μM primer F (forward), R (reverse) were added into the PCR mixture which contain 10 μL 5X PrimeSTAR® buffer (Mg^2+^ plus) (TaKaRa, Dalian, China), 4 μL dNTP mixture (2.5 mM) and 0.5 μL PrimeSTAR®HS DNA polymerase (2.5 U/μL) (TaKaRa), then add ddH_2_O to a total volume of 50 μL. Standard PCR and cloning procedure were used to obtain the mutant clones. All 25 PNGS of wt FE Env were mutated individually to have N to Q/D mutations at the positions shown in Table 1 (positions in HXB2 numbering throughout the manuscript). The entire Env gene of each mutant was sequenced to confirm mutation. The conservation of the 25 PNGS of FE Env with other HIV-1 is shown in Table 1. The primers used for mutagenesis are listed in Additional file 1: Table S1.

### Pseudovirus preparation, infectivity, titration and neutralization assays

Pseudoviruses were produced by co-transfection of 293FT cells (>90% confluency in a 25 cm^2^ rectangular canted neck cell culture flask, Corning, USA) with 5.3 μg pSG3^ΔEnv^ plasmid and 2.7 μg Env-expressing plasmids using the Lipofectamine 2000 reagent (Invitrogen). Supernatants were harvested 48 hr after transfection, filtered (0.45-μm pore size), and stored at −80°C. The concentration of HIV-1 Gag p24 antigen in viral supernatants was measured by enzyme-linked immunosorbent assay (ELISA) (Vironostika HIV-1 antigen micro-ELISA system; bioMérieux, Boxtel, The Netherlands).

A fixed amount of pseudovirus (equivalent to 1.0 ng p24 antigen) was added to TZM-bl cells at 70−80% confluency in a 96-well plate in the presence of 15 μg/mL DEAE-dextran, in a total volume of 200 μL. 48 hr after infection, the luciferase activity in infected cells was measured using the Bright-Glo™ luciferase assay system (Promega, Madison, WI). Relative infectivity was calculated by dividing the Log_10_ (RLU of mutant) by Log_10_ (RLU of wt).

The 50% tissue culture infectious dose (TCID_50_) of a single infectious pseudovirus batch was determined in TZM-bl cells, as described previously [59].

Neutralization was measured as a reduction in luciferase expression after a single-round infection of TZM-bl cells with pseudoviruses according to previously published method [21].

### Gp120 incorporation into virions

293FT cells were co-transfected with plasmid pSG3^ΔEnv^ and Env-expressing plasmids. Viral supernatants were harvested and filtered (0.45-μm pore size) 48 hr post transfection, then were pelleted through a 25% sucrose cushion by ultracentrifugation at 100,000 × g for 2.5 hr. The layers of supernatant and sucrose were removed, and the resulting viral pellets were resuspended in 250 μl PBS. The viral pellets were subjected to p24 ELISA, and gp120 ELISA (Immune Technology Corp, USA) [60-62] to determine the amount of p24 and gp120. Incorporation was determined by calculating the ratio of gp120 to p24. The supernatant of transfected cells by single plasmid pSG3^ΔEnv^ or FE Env-expressing plasmid were set as control, which were performed as the viral supernatant above.

### Structural modeling

The core FE gp120 model was created using the previously solved structure of truncated gp120 from a clade B HIV-1 isolate, JR-FL (PDB: 2B4C), as a template. Based on an alignment using Multalin, deletions were made to the FE gp120 sequence to correspond with the truncations made in the JR-FL sequence of the crystallized construct. The comparative homology modeling program SWISS-MODEL was used to generate the FE gp120 model. The FE V1/V2 domain model was generated similarly using the recently solved structure of the V1/V2 domain from a clade C isolate, CAP4 (PDB: 3U4E) as a template. The FE gp120 model including the V1/V2 domain was created in PyMOL by manually grafting the V1/V2 loop model onto the FE gp120 monomer model based on sequence and structural alignments of the limited overlapping sequences between the two molecules. The model of the FE gp41 was generated using the structure of the gp41 six-helical core bundle of the HXB2 group M subtype B isolate (PDB: 2X7R) as a template. For the “closed” trimer model we first generated an FE monomer model using the unliganded SIV gp120 structure as a template (PDB: 2BF1). The model was generated with Multalin and SWISS MODEL as described above. Our FE unliganded monomer model was then superimposed onto each of the monomers in the “closed” trimer model that was generated from cryo-ET (“closed” state PDB: 3DNN). The FE gp120 trimer model in the “open” CD4 bound state was generated by superimposing our FE gp120 monomer with V1/V2 domain model onto each of the monomers in the CD4 bound trimer model generated from cryo-ET (“open” CD4 bound state PDB: 3DNO).

### Statistical analysis

Error bars indicate the standard deviation of the means calculated by Prism software 5.0. One-way analysis of variance with Tukey’s multiple comparison was used to test whether the mean infectivity and gp120 incorporation is significantly different between wild type and mutants by SPSS 10.0. A *p* value <0.05 was considered significant and is depicted by an asterisk in Figure 3.

## Abbreviations

Env: Envelope glycoprotein; PNGS: Potential N-linked glycosylation site; CRFs: The circulating recombinant forms; TCID_50_: The 50% tissue culture infectious dose; MAb: Monoclonal antibody; MPER: Membrane proximal external region.

## Competing interests

The authors declare that they have no competing interests.

## Authors’ contributions

WW performed the experiments and analyzed data; WWB, XS. C and YC conceived and designed the experiments; JN and SW constructed the wildtype Env expressing plasmid FE; CP, CT and XS. C made the structure modeling; WW, XS. C and YW wrote the paper; all authors read and approved the final document.

## Supplementary Material

Additional file 1: Table S1 List of primers used for site-directed mutagenesis to eliminate potential N-linked glycosylation sites in the FE envelope. Click here for file

## References

[B1] Tan K Liu J Wang J Shen S Lu M Atomic structure of a thermostable subdomain of HIV-1 gp41 Proc Natl Acad Sci U S A 1997 94 12303 12308 9356444 10.1073/pnas.94.23.12303 PMC24915

[B2] Weissenhorn W Dessen A Harrison SC Skehel JJ Wiley DC Atomic structure of the ectodomain from HIV-1 gp41 Nature 1997 387 426 430 9163431 10.1038/387426a0

[B3] Wyatt R Sodroski J The HIV-1 envelope glycoproteins: fusogens, antigens, and immunogens Science 1998 280 1884 1888 9632381 10.1126/science.280.5371.1884

[B4] Bazan HA Alkhatib G Broder CC Berger EA Patterns of CCR5, CXCR4, and CCR3 usage by envelope glycoproteins from human immunodeficiency virus type 1 primary isolates J Virol 1998 72 4485 4491 9557746 10.1128/jvi.72.5.4485-4491.1998 PMC109686

[B5] Bjorndal A Deng H Jansson M Fiore JR Colognesi C Karlsson A Albert J Scarlatti G Littman DR Fenyo EM Coreceptor usage of primary human immunodeficiency virus type 1 isolates varies according to biological phenotype J Virol 1997 71 7478 7487 9311827 10.1128/jvi.71.10.7478-7487.1997 PMC192094

[B6] Freed EO Martin MA The role of human immunodeficiency virus type 1 envelope glycoproteins in virus infection J Biol Chem 1995 270 23883 23886 7592573 10.1074/jbc.270.41.23883

[B7] Kwong PD Wyatt R Robinson J Sweet RW Sodroski J Hendrickson WA Structure of an HIV gp120 envelope glycoprotein in complex with the CD4 receptor and a neutralizing human antibody Nature 1998 393 648 659 9641677 10.1038/31405 PMC5629912

[B8] McLellan JS Pancera M Carrico C Gorman J Julien JP Khayat R Louder R Pejchal R Sastry M Dai K Structure of HIV-1 gp120 V1/V2 domain with broadly neutralizing antibody PG9 Nature 2011 480 336 343 22113616 10.1038/nature10696 PMC3406929

[B9] Chen B Vogan EM Gong H Skehel JJ Wiley DC Harrison SC Structure of an unliganded simian immunodeficiency virus gp120 core Nature 2005 433 834 841 15729334 10.1038/nature03327

[B10] Frey G Peng H Rits-Volloch S Morelli M Cheng Y Chen B A fusion-intermediate state of HIV-1 gp41 targeted by broadly neutralizing antibodies Proc Natl Acad Sci U S A 2008 105 3739 3744 18322015 10.1073/pnas.0800255105 PMC2268799

[B11] Harrison SC Mechanism of membrane fusion by viral envelope proteins Adv Virus Res 2005 64 231 261 16139596 10.1016/S0065-3527(05)64007-9 PMC7173036

[B12] Burton DR Desrosiers RC Doms RW Koff WC Kwong PD Moore JP Nabel GJ Sodroski J Wilson IA Wyatt RT HIV vaccine design and the neutralizing antibody problem Nat Immunol 2004 5 233 236 14985706 10.1038/ni0304-233

[B13] Leonard CK Spellman MW Riddle L Harris RJ Thomas JN Gregory TJ Assignment of intrachain disulfide bonds and characterization of potential glycosylation sites of the type 1 recombinant human immunodeficiency virus envelope glycoprotein (gp120) expressed in Chinese hamster ovary cells J Biol Chem 1990 265 10373 10382 2355006

[B14] Huang X Jin W Hu K Luo S Du T Griffin GE Shattock RJ Hu Q Highly conserved HIV-1 gp120 glycans proximal to CD4-binding region affect viral infectivity and neutralizing antibody induction Virology 2012 423 97 106 22192629 10.1016/j.virol.2011.11.023

[B15] Kumar R Tuen M Li H Tse DB Hioe CE Improving immunogenicity of HIV-1 envelope gp120 by glycan removal and immune complex formation Vaccine 2011 29 9064 9074 21945958 10.1016/j.vaccine.2011.09.057 PMC3328143

[B16] Scanlan CN Pantophlet R Wormald MR Ollmann Saphire E Stanfield R Wilson IA Katinger H Dwek RA Rudd PM Burton DR The broadly neutralizing anti-human immunodeficiency virus type 1 antibody 2G12 recognizes a cluster of alpha1–>2 mannose residues on the outer face of gp120 J Virol 2002 76 7306 7321 12072529 10.1128/JVI.76.14.7306-7321.2002 PMC136327

[B17] Pollakis G Kang S Kliphuis A Chalaby MI Goudsmit J Paxton WA N-linked glycosylation of the HIV type-1 gp120 envelope glycoprotein as a major determinant of CCR5 and CXCR4 coreceptor utilization J Biol Chem 2001 276 13433 13441 11278567 10.1074/jbc.M009779200

[B18] Wei X Decker JM Wang S Hui H Kappes JC Wu X Salazar-Gonzalez JF Salazar MG Kilby JM Saag MS Antibody neutralization and escape by HIV-1 Nature 2003 422 307 312 12646921 10.1038/nature01470

[B19] Myers GRA, Josephs SF, Smith TF, Wong-Staal F, Berzofsky JA Human Retroviruses and AIDS 1989: A Compilation and Analysis of Nucleic Acid and Amino Acid Sequences 1989 Los Alamos, NM: Theoretical Biology and Biophysics Group, Los Alamos National Laboratory

[B20] Wang S Nie J Wang Y Comparisons of the genetic and neutralization properties of HIV-1 subtype C and CRF07/08_BC env molecular clones isolated from infections in China Virus Res 2011 155 137 146 20875470 10.1016/j.virusres.2010.09.012

[B21] Chong H Hong K Zhang C Nie J Song A Kong W Wang Y Genetic and neutralization properties of HIV-1 env clones from subtype B/BC/AE infections in China J Acquir Immune Defic Syndr 2008 47 535 543 18209676 10.1097/QAI.0b013e3181663967

[B22] Nie J Zhang C Liu W Wu X Li F Wang S Liang F Song A Wang Y Genotypic and phenotypic characterization of HIV-1 CRF01_AE env molecular clones from infections in China J Acquir Immune Defic Syndr 2010 53 440 450 20090544 10.1097/QAI.0b013e3181cb8300

[B23] Buzon V Natrajan G Schibli D Campelo F Kozlov MM Weissenhorn W Crystal structure of HIV-1 gp41 including both fusion peptide and membrane proximal external regions PLoS Pathog 2010 6 e1000880 20463810 10.1371/journal.ppat.1000880 PMC2865522

[B24] Kolchinsky P Kiprilov E Bartley P Rubinstein R Sodroski J Loss of a single N-linked glycan allows CD4-independent human immunodeficiency virus type 1 infection by altering the position of the gp120 V1/V2 variable loops J Virol 2001 75 3435 3443 11238869 10.1128/JVI.75.7.3435-3443.2001 PMC114136

[B25] Liu J Bartesaghi A Borgnia MJ Sapiro G Subramaniam S Molecular architecture of native HIV-1 gp120 trimers Nature 2008 455 109 113 18668044 10.1038/nature07159 PMC2610422

[B26] White TA Bartesaghi A Borgnia MJ Meyerson JR de la Cruz MJ Bess JW Nandwani R Hoxie JA Lifson JD Milne JL Subramaniam S Molecular architectures of trimeric SIV and HIV-1 envelope glycoproteins on intact viruses: strain-dependent variation in quaternary structure PLoS Pathog 2010 6 e1001249 21203482 10.1371/journal.ppat.1001249 PMC3009598

[B27] Rusert P Krarup A Magnus C Brandenberg OF Weber J Ehlert AK Regoes RR Gunthard HF Trkola A Interaction of the gp120 V1V2 loop with a neighboring gp120 unit shields the HIV envelope trimer against cross-neutralizing antibodies J Exp Med 2011 208 1419 1433 21646396 10.1084/jem.20110196 PMC3135368

[B28] Harris A Borgnia MJ Shi D Bartesaghi A He H Pejchal R Kang YK Depetris R Marozsan AJ Sanders RW Trimeric HIV-1 glycoprotein gp140 immunogens and native HIV-1 envelope glycoproteins display the same closed and open quaternary molecular architectures Proc Natl Acad Sci U S A 2011 108 11440 11445 21709254 10.1073/pnas.1101414108 PMC3136299

[B29] Huang CC Tang M Zhang MY Majeed S Montabana E Stanfield RL Dimitrov DS Korber B Sodroski J Wilson IA Structure of a V3-containing HIV-1 gp120 core Science 2005 310 1025 1028 16284180 10.1126/science.1118398 PMC2408531

[B30] Hartley O Klasse PJ Sattentau QJ Moore JP V3: HIV’s switch-hitter AIDS Res Hum Retroviruses 2005 21 171 189 15725757 10.1089/aid.2005.21.171

[B31] Chen M Shi C Kalia V Tencza SB Montelaro RC Gupta P HIV gp120 V(1)/V(2) and C(2)-V(3) domains glycoprotein compatibility is required for viral replication Virus Res 2001 79 91 101 11551649 10.1016/s0168-1702(01)00322-7

[B32] Francois KO Balzarini J The highly conserved glycan at asparagine 260 of HIV-1 gp120 is indispensable for viral entry J Biol Chem 2011 286 42900 42910 22006924 10.1074/jbc.M111.274456 PMC3234821

[B33] Pancera M Majeed S Ban YE Chen L Huang CC Kong L Kwon YD Stuckey J Zhou T Robinson JE Structure of HIV-1 gp120 with gp41-interactive region reveals layered envelope architecture and basis of conformational mobility Proc Natl Acad Sci U S A 2010 107 1166 1171 20080564 10.1073/pnas.0911004107 PMC2824281

[B34] Helseth E Olshevsky U Furman C Sodroski J Human immunodeficiency virus type 1 gp120 envelope glycoprotein regions important for association with the gp41 transmembrane glycoprotein J Virol 1991 65 2119 2123 2002555 10.1128/jvi.65.4.2119-2123.1991 PMC240081

[B35] Leavitt M Park EJ Sidorov IA Dimitrov DS Quinnan GV Jr Concordant modulation of neutralization resistance and high infectivity of the primary human immunodeficiency virus type 1 MN strain and definition of a potential gp41 binding site in gp120 J Virol 2003 77 560 570 12477860 10.1128/JVI.77.1.560-570.2003 PMC140585

[B36] Sen J Jacobs A Caffrey M Role of the HIV gp120 conserved domain 5 in processing and viral entry Biochemistry 2008 47 7788 7795 18597484 10.1021/bi800227z

[B37] Thali M Furman C Helseth E Repke H Sodroski J Lack of correlation between soluble CD4-induced shedding of the human immunodeficiency virus type 1 exterior envelope glycoprotein and subsequent membrane fusion events J Virol 1992 66 5516 5524 1501286 10.1128/jvi.66.9.5516-5524.1992 PMC289110

[B38] Wang J Sen J Rong L Caffrey M Role of the HIV gp120 conserved domain 1 in processing and viral entry J Biol Chem 2008 283 32644 32649 18815131 10.1074/jbc.M806099200 PMC2583318

[B39] Yang X Mahony E Holm GH Kassa A Sodroski J Role of the gp120 inner domain beta-sandwich in the interaction between the human immunodeficiency virus envelope glycoprotein subunits Virology 2003 313 117 125 12951026 10.1016/s0042-6822(03)00273-3

[B40] Go EP Chang Q Liao HX Sutherland LL Alam SM Haynes BF Desaire H Glycosylation site-specific analysis of clade C HIV-1 envelope proteins J Proteome Res 2009 8 4231 4242 19610667 10.1021/pr9002728 PMC2756219

[B41] Pabst M Chang M Stadlmann J Altmann F Glycan profiles of the 27 N-glycosylation sites of the HIV envelope protein CN54gp140 Biol Chem 2012 393 719 730 22944675 10.1515/hsz-2012-0148

[B42] Lavine CL Lao S Montefiori DC Haynes BF Sodroski JG Yang X High-mannose glycan-dependent epitopes are frequently targeted in broad neutralizing antibody responses during human immunodeficiency virus type 1 infection J Virol 2012 86 2153 2164 22156525 10.1128/JVI.06201-11 PMC3302386

[B43] Pejchal R Wilson IA Structure-based vaccine design in HIV: blind men and the elephant? Curr Pharm Des 2010 16 3744 3753 21128885 10.2174/138161210794079173 PMC3096478

[B44] Saphire EO Parren PW Pantophlet R Zwick MB Morris GM Rudd PM Dwek RA Stanfield RL Burton DR Wilson IA Crystal structure of a neutralizing human IGG against HIV-1: a template for vaccine design Science 2001 293 1155 1159 11498595 10.1126/science.1061692

[B45] Li Y Cleveland B Klots I Travis B Richardson BA Anderson D Montefiori D Polacino P Hu SL Removal of a single N-linked glycan in human immunodeficiency virus type 1 gp120 results in an enhanced ability to induce neutralizing antibody responses J Virol 2008 82 638 651 17959660 10.1128/JVI.01691-07 PMC2224603

[B46] Pantophlet R Ollmann Saphire E Poignard P Parren PW Wilson IA Burton DR Fine mapping of the interaction of neutralizing and nonneutralizing monoclonal antibodies with the CD4 binding site of human immunodeficiency virus type 1 gp120 J Virol 2003 77 642 658 12477867 10.1128/JVI.77.1.642-658.2003 PMC140633

[B47] Utachee P Nakamura S Isarangkura-Na-Ayuthaya P Tokunaga K Sawanpanyalert P Ikuta K Auwanit W Kameoka M Two N-linked glycosylation sites in the V2 and C2 regions of human immunodeficiency virus type 1 CRF01_AE envelope glycoprotein gp120 regulate viral neutralization susceptibility to the human monoclonal antibody specific for the CD4 binding domain J Virol 2010 84 4311 4320 20164234 10.1128/JVI.02619-09 PMC2863754

[B48] Wu X Zhou T Zhu J Zhang B Georgiev I Wang C Chen X Longo NS Louder M McKee K Focused evolution of HIV-1 neutralizing antibodies revealed by structures and deep sequencing Science 2011 333 1593 1602 21835983 10.1126/science.1207532 PMC3516815

[B49] Zhou T Georgiev I Wu X Yang ZY Dai K Finzi A Kwon YD Scheid JF Shi W Xu L Structural basis for broad and potent neutralization of HIV-1 by antibody VRC01 Science 2010 329 811 817 20616231 10.1126/science.1192819 PMC2981354

[B50] Zhou T Xu L Dey B Hessell AJ Van Ryk D Xiang SH Yang X Zhang MY Zwick MB Arthos J Structural definition of a conserved neutralization epitope on HIV-1 gp120 Nature 2007 445 732 737 17301785 10.1038/nature05580 PMC2584968

[B51] Muster T Steindl F Purtscher M Trkola A Klima A Himmler G Ruker F Katinger H A conserved neutralizing epitope on gp41 of human immunodeficiency virus type 1 J Virol 1993 67 6642 6647 7692082 10.1128/jvi.67.11.6642-6647.1993 PMC238102

[B52] Zwick MB Labrijn AF Wang M Spenlehauer C Saphire EO Binley JM Moore JP Stiegler G Katinger H Burton DR Parren PW Broadly neutralizing antibodies targeted to the membrane-proximal external region of human immunodeficiency virus type 1 glycoprotein gp41 J Virol 2001 75 10892 10905 11602729 10.1128/JVI.75.22.10892-10905.2001 PMC114669

[B53] Ma BJ Alam SM Go EP Lu X Desaire H Tomaras GD Bowman C Sutherland LL Scearce RM Santra S Envelope deglycosylation enhances antigenicity of HIV-1 gp41 epitopes for both broad neutralizing antibodies and their unmutated ancestor antibodies PLoS Pathog 2011 7 e1002200 21909262 10.1371/journal.ppat.1002200 PMC3164629

[B54] Walker LM Phogat SK Chan-Hui PY Wagner D Phung P Goss JL Wrin T Simek MD Fling S Mitcham JL Broad and potent neutralizing antibodies from an African donor reveal a new HIV-1 vaccine target Science 2009 326 285 289 19729618 10.1126/science.1178746 PMC3335270

[B55] Pejchal R Doores KJ Walker LM Khayat R Huang PS Wang SK Stanfield RL Julien JP Ramos A Crispin M A potent and broad neutralizing antibody recognizes and penetrates the HIV glycan shield Science 2011 334 1097 1103 21998254 10.1126/science.1213256 PMC3280215

[B56] Sanders RW Venturi M Schiffner L Kalyanaraman R Katinger H Lloyd KO Kwong PD Moore JP The mannose-dependent epitope for neutralizing antibody 2G12 on human immunodeficiency virus type 1 glycoprotein gp120 J Virol 2002 76 7293 7305 12072528 10.1128/JVI.76.14.7293-7305.2002 PMC136300

[B57] Binley JM Wrin T Korber B Zwick MB Wang M Chappey C Stiegler G Kunert R Zolla-Pazner S Katinger H Comprehensive cross-clade neutralization analysis of a panel of anti-human immunodeficiency virus type 1 monoclonal antibodies J Virol 2004 78 13232 13252 15542675 10.1128/JVI.78.23.13232-13252.2004 PMC524984

[B58] Marshall RD Glycoproteins Annu Rev Biochem 1972 41 673 702 4563441 10.1146/annurev.bi.41.070172.003325

[B59] Li M Gao F Mascola JR Stamatatos L Polonis VR Koutsoukos M Voss G Goepfert P Gilbert P Greene KM Human immunodeficiency virus type 1 env clones from acute and early subtype B infections for standardized assessments of vaccine-elicited neutralizing antibodies J Virol 2005 79 10108 10125 16051804 10.1128/JVI.79.16.10108-10125.2005 PMC1182643

[B60] Bhakta SJ Shang L Prince JL Claiborne DT Hunter E Mutagenesis of tyrosine and di-leucine motifs in the HIV-1 envelope cytoplasmic domain results in a loss of Env-mediated fusion and infectivity Retrovirology 2011 8 37 21569545 10.1186/1742-4690-8-37 PMC3117779

[B61] Hammonds J Chen X Ding L Fouts T De Vico A zur Megede J Barnett S Spearman P Gp120 stability on HIV-1 virions and Gag-Env pseudovirions is enhanced by an uncleaved Gag core Virology 2003 314 636 649 14554091 10.1016/s0042-6822(03)00467-7

[B62] Shang L Yue L Hunter E Role of the membrane-spanning domain of human immunodeficiency virus type 1 envelope glycoprotein in cell-cell fusion and virus infection J Virol 2008 82 5417 5428 18353944 10.1128/JVI.02666-07 PMC2395181

