# Supplementary material for: A systematic study of the N-glycosylation sites of HIV-1 envelope protein on infectivity and antibody-mediated neutralization
Source: Retrovirology. 2013 Feb 6;10:14. doi: 10.1186/1742-4690-10-14 (PMC3648360; doi:10.1186/1742-4690-10-14)
Supplement: Additional file 1: Table S1 — List of primers used for site-directed mutagenesis to eliminate potential N-linked glycosylation sites in the FE envelope. [file 1742-4690-10-14-S1.doc]

Table S1. List of primers used for site-directed mutagenesis to eliminate potential N-linked glycosylation sites in the FE envelope.

| Primer | Sequence^a^ |
| --- | --- |
| N88Q | F: CAAGAAATAGTTTTGAGACAGGTAACAGAAAATTTTAAC |
|  | R: GTTAAAATTTTCTGTTACCTGTCTCAAAACTATTTCTTG |
| N88D | F: CAAGAAATAGTTTTGAGAGATGTAACAGAAAATTTTAAC |
|  | R: GTTAAAATTT TCTGTTACATCTCTCAAAAC TATTTCTTG |
| N133Q | F: CACTTTAGAATGTAATCAGGTTACCAAAAATGAG |
|  | R: CTCATTTTTGGTAACCTGATTACATTCTAAAGTG |
| N142Q | F: GTAATAATGTTACCAAACAGGAGACCTACCATGAGAG |
|  | R: CTCTCATGGTAGGTCTCCTGTTTGGTAACATTATTAC |
| N156Q | F: GAAT GAAGGAAATGAAACAGTGCTCTTTCAATGCAAC |
|  | R: GTTGCATTGAAAGAGCACTGTTTCATTTCCTTCATTC |
| N156D | F: GAATGAAGGAAATGAAAGATTGCTCTTTCAATGCAAC |
|  | R: GTTGCATTGAAAGAGCAATCTTTCATTTCCTTCATTC |
| N156S | F: GAAGGAAATGAAAAGTTGCTCTTTCAATG |
|  | R: CATTGAAAGAGCAACTTTTCATTTCCTTC |
| N160Q | F: GAAAAATTGCTCTTTCCAGGCAACCACAGAATTAAG |
|  | R: CTTAATTCTGTGGTTGCCTGGAAAGAGCAATTTTTC |
| N160D | F: GAAAAATTGCTCTTTCGATGCAACCACAGAATTAAG |
|  | R: CTTAATTCTGTGGTTGCATCGAAAGAGCAA TTTTTC |
| N181Q | F: CACTTAATTCTATTGATCAGTCTAGTAAGTATTATAG |
|  | R: CTATAATACTTACTAGACTGATCAATAGAATTAAGTG |
| N181D | F: CACTTAATTCTATTGATGACTCTAGTAAGTATTATAG |
|  | R: CTATAATACT TACTAGAGTCATCAATAGAA TTAAGTG |
| N197Q | F: GATTAATAAATTGTCAGACCTCAGCCATAACAC |
|  | R: GTGTTATGGCTGAGGTCTGACAATTTATTAATC |
| N197D | F: GATTAATAAATTGTGATACCTCAGCCATAACAC |
|  | R: GTGTTATGGCTGAGGTATCACAATTTATTAATC |
| N234Q | F: GTAATGATAAGATATTCCAGGGGACAGGACCATGCC |
|  | R: GGCATGGTCCTGTCCCCTGGAATATCTTATCATTAC |
| N234D | F: GTAATGATAAGATATTCGATGGGACAGGACCATGCC |
|  | R: GGCATGGTCC TGTCCCATCG AATATCTTAT CATTAC |
| N241Q | F: GACAGGACCATGCCATCAGGTTAGTACCGTGCAATG |
|  | R: CATTGCACGGTACTAACCTGATGGCATGGTCCTGTC |
| N241D | F: GACAGGACCATGCCATGATGTTAGTACCGTGCAATG |
|  | R: CATTGCACGG TACTAACATC ATGGCATGGT CCTGTC |
| N262Q | F: CAACTCAACTACTGTTACAGGGTAGCATAGCAGAAG |
|  | R: CTTCTGCTATGCTACCCTGTAACAGTAGTTGAGTTG |
| N262D | F: CAACTCAACTACTGTTAGATGGTAGCATAGCAGAAG |
|  | R: CTTCTGCTATGCTACCATCTAACAGTAGTTGAGTTG |
| N289Q | F: CAATAATAGTACATCTTCAGCAATCTGTAGAAATTATATG |
|  | R: CATATAATTTCTACAGATTGCTGAAGATGTACTATTATTG |
| N289D | F: CAATAATAGTACATCTTGATCAATCTGTAGAAATTATATG |
|  | R: CATATAATTT CTACAGATTGATCAAGATGTACTATTATTG |
| N301Q | F: GTACAAGACCTGGCCAGAATACAAGAAAAAG |
|  | R: CTTTTTCTTGTATTCTGGCCAGGTCTTGTAC |
| N339Q | F: CATTAATAAAATTAAATGGCAGGAAACTTTACAAAGGGTAAG |
|  | R: CTTACCCTTTGTAAAGTTTCCTGCCATTTAATTTTATTAATG |
| N355Q | F: GCAGAACACTTCCAGCAGAAAACAATAAAATTTG |
|  | R: CAAATTTTATTGTTTTCTGCTGGAAGTGTTCTGC |
| N392Q | F: CATCAGGCCTGTTTCAGGAGACATACATGTC |
|  | R: GACATGTATGTCTCCTGAAACAGGCCTGATG |
| N408Q | F: GAGACATACATGTCTCAGGGTTCAAACTCAACC |
|  | R: GGTTGAGTTTGAACCCTGAG ACATGTATGTCTC |
| N411Q | F: GTCTAATGGTTCACAGTCAACCATCACAATC |
|  | R: GATTGTGATGGTTGACTGTGAACCATTAGAC |
| N442Q | F: CCCATTGAAGGACAGATAACATGTAAATC |
|  | R: GATTTACATGTTATCTGTCCTTCAATGGG |
| N448Q | F: CATAACATGTAAATCACAGATCACAGGACTAC |
|  | R: GTAGTCCTGTGATCTGTGATTTACATGTTATG |
| N463Q | F: GATGGAGGACTAGCACAGGATACAAATGGTACAG |
|  | R: CTGTACCATTTGTATCCTGTGCTAGTCCTCCATC |
| N466Q | F: CTAGCAAATGATACACAGGGTACAGAGACATTC |
|  | R: GAATGTCTCTGTACCCTGTGTATCATTTGCTAG |
| N611Q | F: CTGCTGTGCCTTGGCAGTCCAGTTGGAGTAAC |
|  | R: GTTACTCCAACTGGACTGCCAAGGCACAGCAG |
| N616Q | F: CTCCAGTTGGAGTCAGAAAACTCAAAATG |
|  | R: CATTTTGAGTTTTCTGACTCCAACTGGAG |
| N616D | F: CTCCAGTTGGAGTGACAAAACTCAAAATG |
|  | R: CATTTTGAGTTTTGTCACTCCAACTGGAG |
| N625Q | F: GAGATTTGGGATCAGATGACCTGGATGC |
|  | R: GCATCCAGGTCATCTGATCCCAAATCTC |
| N637Q | F: GGGATAAAGAAATTAGTCAGTACACAAACACAATATAC |
|  | R: GTATATTGTGTTTGTGTACTGACTAATTTCTTTATCCC |

^a^ F, forward primer; R, reverse primer. Underlining indicates nucleotides to change
